# Characterization of kinesin switch I mutations that cause hereditary spastic paraplegia

**DOI:** 10.1371/journal.pone.0180353

**Published:** 2017-07-05

**Authors:** Scott Jennings, Madeline Chenevert, Liqiong Liu, Madhusoodanan Mottamal, Edward J. Wojcik, Thomas M. Huckaba

**Affiliations:** 1 Department of Biology, Xavier University of Louisiana, New Orleans, Louisiana, United States of America; 2 Department of Biochemistry and Molecular Biology, LSU School of Medicine & Health Sciences Center, New Orleans, Louisiana, United States of America; 3 RCMI Molecular Modeling Core, Xavier University of Louisiana, New Orleans, Louisiana, United States of America; University of Toronto, CANADA

## Abstract

Kif5A is a neuronally-enriched isoform of the Kinesin-1 family of cellular transport motors. 23 separate mutations in the motor domain of Kif5A have been identified in patients with the complicated form of hereditary spastic paraplegia (HSP). We performed in vitro assays on dimeric recombinant Kif5A with HSP-causing mutations in the Switch I domain, which participates in the coordination and hydrolysis of ATP by kinesin. We observed a variety of significantly reduced catalytic and mechanical activities as a result of each mutation, with the shared phenotype from each that motility was significantly reduced. Substitution of Mn^2+^ for Mg^2+^ in our reaction buffers provides a dose-dependent rescue in both the catalytic and ensemble mechanical properties of the S203C mutant. This work provides mechanistic insight into the cause of HSP in patients with these mutations and points to future experiments to further dissect the root cause of this disease.

## Introduction

Hereditary Spastic Paraplegia (HSP) is a genetically and clinically heterogeneous disease that involves the progressive degeneration of axons in the corticospinal tract [1–3]. To date, 76 distinct genetic loci and 59 separate human genes have been implicated in the onset of HSP [4]. HSP manifests in either the simple or complicated form of the disease. While progressive lower limb spasticity is common in both forms, patients with the complicated form of HSP may also present with retinopathy, ataxia, peripheral polyneuropathy, and cognitive deficit [5, 6]. Approximately 10% of the known cases of complicated HSP are caused by mutations in the neuronally-enriched, kinesin-1 family member Kif5A [6].

Of the 25 HSP-causing mutations in Kif5A that have been published to date, 23 map to the motor domain of the protein (Table 1) [6–19]. The kinesin motor domain contains the microtubule binding site, as well as the nucleotide catalytic site. Together with the neck linker, the motor domain is responsible for force generation necessary for the motility of cellular cargoes [20]. Nucleotide catalysis (binding, hydrolysis, and product release) is carried out by three conserved motifs of the motor domain known as the P-loop, Switch I, and Switch II [21]. The conservation of these three motifs in metazoan kinesins is such that computational algorithms use their consensus sequences for kinesin gene prediction. Thus, it is unsurprising that mutations in these conserved regions would give rise to human disease.

**Table 1:** Kif5A mutations that cause HSP.

Mutation	Cause	Domain	Ref.
Y63C	A188G	α1	[6]
D73N	G217A	α1	[18]
R162W	C484T	L7	[8]
M198T	T593C	L9/Switch I	[6]
S202N	G605A	Switch I	[9]
S203C	C608G	Switch I	[15]
R204W	C610T	Switch I	[19]
R204Q	G611A	Switch I	[6]
V231L	G691T	β7/Switch II	[9]
D232N	G694A	Switch II	[11]
E251K	G751A	L11	[6]
K253N	G759T	L11	[18]
N256S	A767G	L11	[16]
ΔN256	Δ768–770	L11	[18]
K257N	G771C	α4	[6]
S258L	C773T	α4	[13]
L259Q	T776A	α4	[14]
Y276C	A827G	L12	[7]
P278L	C833T	L12	[13]
R280C	C838T	L12	[10]
R280H	G839A	L12	[6]
R280L	G839T	L12	[6]
R323W	C967T	α6	[17]
A361V	G1081T	Stalk	[12]
E755K	G2263A	Stalk	[9]

Mutations described in the literature were mapped onto the human Kif5A sequence in NCBI (Gene ID: 3798) with the start codon representing positions 1–3 in the genomic sequence. Domain identification was determined by locating the amino acid in the founder kinesin solution structure 1BG2 [22].

This study focuses on the four Kif5A mutations in Switch I, as well as an additional mutation immediately adjacent to Switch I, that cause HSP. Switch I residues have the dual function of coordinating the two water molecules involved in nucleophilic attack of the γ-phosphate bond, as well as coordinating the Mg^2+^ ion necessary for charge stabilization during hydrolysis [23]. In addition, the terminal arginine of the Switch I consensus (NXXSSR) forms a salt bridge with a glutamate in Switch II that is believed to be essential for ATP hydrolysis [24, 25]. Crystal structures of Kif5B (a Kif5A ortholog showing 85.4% identity and 91.7% conservation in the motor domain) in various nucleotide states shows that Switch I is unstructured in the nucleotide-free and ATP bound-like states, but undergoes a transition to an ordered structure when ADP is bound [22, 26, 27]. This presents the possibility that conserved Switch I residues may have separable roles at different stages of the catalytic cycle and that loss of these roles may impact kinesin function in the context of HSP.

Here we identify the catalytic deficits caused by HSP-causing mutations in Switch I of Kif5A by testing the biochemical and biophysical properties of recombinantly-expressed human Kif5A. As predicted, ATPase rates are significantly reduced in each, although there is a continuum of function loss. In addition, we characterize a potential mechanotransduction mechanism between the microtubule binding domain and the nucleotide binding pocket. Finally, we show evidence for a dose-dependent effect of alternative divalent cation on the activity of one of the HSP mutants.

## Materials and methods

### Protein expression and purification

The first 560 amino acids of human Kif5A were subcloned from a cDNA plasmid obtained from ATCC (Image ID 40148192) into a bacterial expression vector in front of EGFP and a C-terminal 6xHIS tag. This sequence was chosen to correspond to the well-characterized K560 construct of mammalian Kif5B [28]. Site-directed mutagenesis was performed to insert the mutations as indicated. All constructs were sequenced to ensure proper nucleotide sequence and frame. Proteins were expressed and purified as described previously [29], with the exception of an additional gel filtration step following elution from the Ni-NTA column. The imidazole-washed Ni-NTA eluate was concentrated to a volume of 500 μL and separated over a Superdex 200 Increase 10/300 GL column (GE Healthcare) that was pre-equilibrated to gel filtration buffer (25 mM PIPES (pH 6.8), 50 mM KCl, 2 mM MgCl_2_, 1 mM EGTA, 1 mM DTT, 1 mM ATP). Kinesin-positive fractions from the gel filtration column were identified by Western Blot. For S203C protein purified only in the presence of Mn^2+^, 2mM MgCl_2_ was replaced in all purification buffers with 2 mM MnCl_2_. Bovine brain tubulin was purified as described previously [30].

### ATPase assays

#### Basal ATPase assays

Kinesins were combined with Na-ATP in TAMx buffers containing 250 mM Tris-acetate (pH = 7.4) and either 2 mM MgCl_2_ (TAMg), 1 mM MgCl_2_ and 1 mM MnCl_2_ (TAMgMn), or 2 mM MnCl_2_ (TAMn). Reagents were combined in a total volume of 60 μL in wells of a clear, flat-bottomed, 96-well half-area microplate (n = 3 for each combination of kinesin and TAMx) and allowed to incubate at room temperature for 10 minutes. Final reagent concentrations in each well were: 500 nM kinesin, and 125 μM Na-ATP. Following the 10-minute incubation, 15 μL of Malachite Green (MG) phosphate assay reagent (BioAssay Systems, POMG-25) was added to each well. Wells were allowed to incubate at room temperature for a further 40 minutes in a SpectraMax M2e microplate reader (Molecular Devices), with OD620 readings taken every 5 minutes to ensure that the absorbance in each well was allowed to plateau during the 10-minute incubation period. The final phosphate concentration for each well was determined using a phosphate standard curve, and the ATPase rate for each kinesin/TAMx combination was calculated from these phosphate concentrations. To account for background signal due to contaminating phosphate in the solutions, identical wells were set up in the plate and the reaction was immediately stopped and measured for signal with the MG reagent. The resulting (background) values were then subtracted from the experimental values to give a more precise analysis of the rate of phosphate formation during the assay.

#### MT-stimulated ATPase assays

Microtubule-stimulated ATPase assays were performed by altering the protocol for basal ATPase assays by 1) reducing the kinesin concentration to 50 nM, 2) increasing the Na-ATP concentration to 250 μM, 3) adding taxol-stabilized microtubules polymerized from 2 μM tubulin (unless stated differently), and 4) decreasing the incubation time to 5 minutes before the addition of the MG reagent. Similar background subtraction and phosphate standard curve corrections were performed.

### Microtubule affinity assays

1 μM Kif5A protein in gel filtration buffer was added to microtubules at a final concentration of 10 μM tubulin with 20 μM taxol for the ATP experiments, while a variety of concentrations of tubulin was used for the AMPPNP experiments (0, 0.1, 0.2, 0.3, 0.4, 0.5, 0.75, 1, 1.5, 2, 4, and 8 μM for WT, and 0, 0.2, 0.4, 0.6, 0.8, 1, 1.5, 2, 4, 8, and 20 μM for the mutants). Either ATP or AMPPNP was added at a final concentration of 5 mM and the solution was incubated at room temperature for 15 minutes. After incubation, the solution was layered on top of an equal volume of 60% glycerol in gel filtration buffer with 20 μM taxol in ultracentrifuge tubes. The samples were then spun in a TLA120.1 rotor in a Beckman tabletop ultracentrifuge at 45,000 RPM for 10 minutes. The supernatant was carefully removed and the pellet was resuspended in an equal volume of gel filtration buffer. The resulting supernatant and pellet fractions were run side-by-side on a gel, transferred to nitrocellulose, and probed with an antibody for tubulin and GFP (on the C-terminus of kinesin). The resulting film was scanned and band densitometry was performed using ImageJ. Results show the fraction Kif5A bound, obtained by dividing the grayscale intensity of the pellet band by the sum of the grayscale intensities of the pellet and supernatant bands.

### Molecular dynamics simulations

All the MD simulations were carried out with NAMD 2.7 simulation package [31]. The initial structure for the kinesin motor domain was taken from the crystal structure of kinesin motor domain in complex with tubulin and darpin (PDB ID: 4HNA) [27]. The same structure was used for creating the initial structures for the three mutant forms of kinesin with E251K, R204Q and R204W point mutations by changing the respective amino acids. Nucelotide and Mg^2+^ ion bound to kinesin were retained in the structure for the simulation studies. After adding the missing hydrogen atoms, the starting structures were explicitly solvated in a rectangular box of TIP3P water molecules with a minimal distance of 10 Å from the protein to the edges of the box. Counter ions were added to neutralize uncompensated charges and salt was added (Na^+^ and Cl^-^ ions) to represent 0.15 M ionic concentration to mimic physiological conditions. For all the calculations the potential parameters for kinesin and nucleotide were taken from the CHARMM27 force field [32]. After each system was set up, they were then subjected to a series of energy minimization and equilibration processes. First, each system was subjected to 1000 steps of steepest descent minimization. All three systems were then equilibrated in three stages. In the first stage, starting with the minimized structure, each system was slowly heated to room temperature in a stepwise manner over a period of 120 ps with the Cα atoms constrained with harmonic forces. Next, each system was then subjected to Langevin dynamics with a damping coefficient of 10 ps^-1^ for 150 ps. Finally, a 250 ps MD simulations at constant pressure and temperature without imposing any restraints on the system were done for each system. Pressure was set at 1 bar using the Nose-Hover Langevin dynamics with a piston period of 200 fs and a piston decay of 100 fs, and the temperature was maintained at 300 K using the Langevin thermostat. Equations of motion were integrated with a time step of 1 fs. Long range electrostatic interactions were computed by the particle mesh Ewald method with a grid density of 1 Å^-1^, and periodic boundary conditions were applied in three directions. After the equilibration, the production run for each system was carried out for 10 ns at constant temperature and pressure with reduced Langevin damping constant (1 ps^-1^) and slower piston decay time (500 fs). The trajectories were saved at 2 ps intervals for the analysis of structural changes upon point mutations in kinesin.

### In vitro motility assays

For microtubule gliding assays, measurements were performed in a ~10 μL flow cell constructed by placing two strips of double-sided sticky tape between a standard glass microscope slide and a plasma-cleaned, 22 mm x 22 mm glass coverslip. Kinesin protein was diluted to 50 nM in motor buffer (25 mM PIPES, pH 6.8; 50 mM NaCl; 1 mM EGTA; 20 μM taxol; 1 mM DTT; and 2 mM XCl_2_, where X is either Mg^2+^, Mn^2+^ or a mixture of Mg^2+^ and Mn^2+^ at a total concentration of 2 mM), added to the flow chamber, and allowed to adhere to the glass surface for 5 minutes. Flowchambers were then washed with motor buffer with 1 mg/mL casein and allowed to incubate for 5 minutes to block non-specific binding to the coverslip. Flowchambers were washed an additional two times with motor buffer with casein. Finally, 20 μL of motility buffer (motor buffer with 1 mg/ml casein, 2 mM ATP, oxygen scavenger (glucose catalase, glucose oxidase, and glucose), an ATP-regenerating system (pyruvate kinase and phosphoenolpyruvate), and rhodamine-labeled microtubules) was added. Time-lapse imaging was performed on an inverted Olympus IX-71 microscope equipped with a Coolsnap CCD camera driven by Micro-Manager open source microscopy software (www.micro-manager.org). To determine the microtubule gliding velocity, the leading edge of a microtubule was tracked through a minimum of 10 successive frames, and the average instantaneous velocity (the sum of the distances traveled in each successive frame divided by the number of frames multiplied by the time between each frame) was obtained using ImageJ. The average for each construct in each condition (n = minimum of 50 microtubules) was determined by applying a simple one-peak Gaussian fit to a histogram of the velocities using Origin 8 software (OriginLab Corporation). Values reported in bar graphs are averages +/- SD. Kymograph images shown in S1 Fig were generated using the Reslice feature of ImageJ by drawing a line along the trace of a single microtubule in the maximum Z-projection from a time-lapse image stack. The slope of the subsequent line (either the end of a microtubule or a fiduciary mark along its length) provides a visual representation of microtubule velocity. Steeper slopes correspond to higher velocities.

### Statistical analysis

All quantitative data are reported as the mean value plus or minus one standard deviation. A Student’s t-test was performed to determine if there was a significant difference in mean values in compared groups. Significance was determined as p<0.05.

## Results

### HSP mutants have reduced ATPase activity

To determine the impact of HSP-causing mutations in Switch I on its enzymatic rate, purified kinesin proteins were subjected to ATPase assays both in the absence (basal) and presence (MT-stimulated) of MTs. As shown previously, adding MTs causes a significant increase in ATPase activity as a result of increasing the rate of ADP release [33–35] (Table 2). All Switch I mutants tested had a significant decrease in the basal ATPase rate. Likewise, the MT-stimulated ATPase rate of all mutants was significantly reduced in the HSP mutants. Since the mutations are in the nucleotide binding pocket (with the exception of E251K), one would expect that the basal ATPase rate would be affected, since the only requirement for basal activity is having the core catalytic domain intact. However, we do note that the relative change between basal and MT-stimulated ATPase rates in some mutants is similar to that of wild type. The catalytic rate of the wild type protein increases > 30-fold upon the addition of microtubules. Likewise, we see a similar increase in catalytic rate in the M198T and S203C mutants. This suggests that the mechanotransduction network of communication induced by the interaction with the microtubule is still intact in these mutants, while in the others (S202N, R204Q/W, and E251K) this has been lost.

**Table 2:** Effects of HSP mutations on the basal and MT-Stimulated ATPase rate.

Kif5A	Basal ATPase Rate (ATP/head/sec)	MT-Stimulated ATPase Rate (ATP/head/sec)	-fold rate increase by MTs
Wild Type	0.204 +/- 0.003	6.77 +/- 0.65	33.2
M198T	0.043 +/- 0.018 *	1.83 +/- 0.43 *	42.6
S202N	0.065 +/- 0.054 *	0.35 +/- 0.08 *	5.4
S203C	0.085 +/- 0.008 *	2.00 +/- 0.33 *	23.5
R204Q	0.047 +/- 0.038 *	0.50 +/- 0.11 *	10.6
R204W	0.039 +/- 0.104 *	-0.22 +/- 0.08 *	-
E251K	0.142 +/- 0.211	0.88 +/- 0.26 *	6.2

Data shown are the average rate for at least three sample runs performed from a minimum of two different protein preparations.

* indicates rates that are significantly lower than the corresponding wild type rate (p<0.01).

### Three of the HSP mutants may have a new rate-limiting step

**Fig 1:**
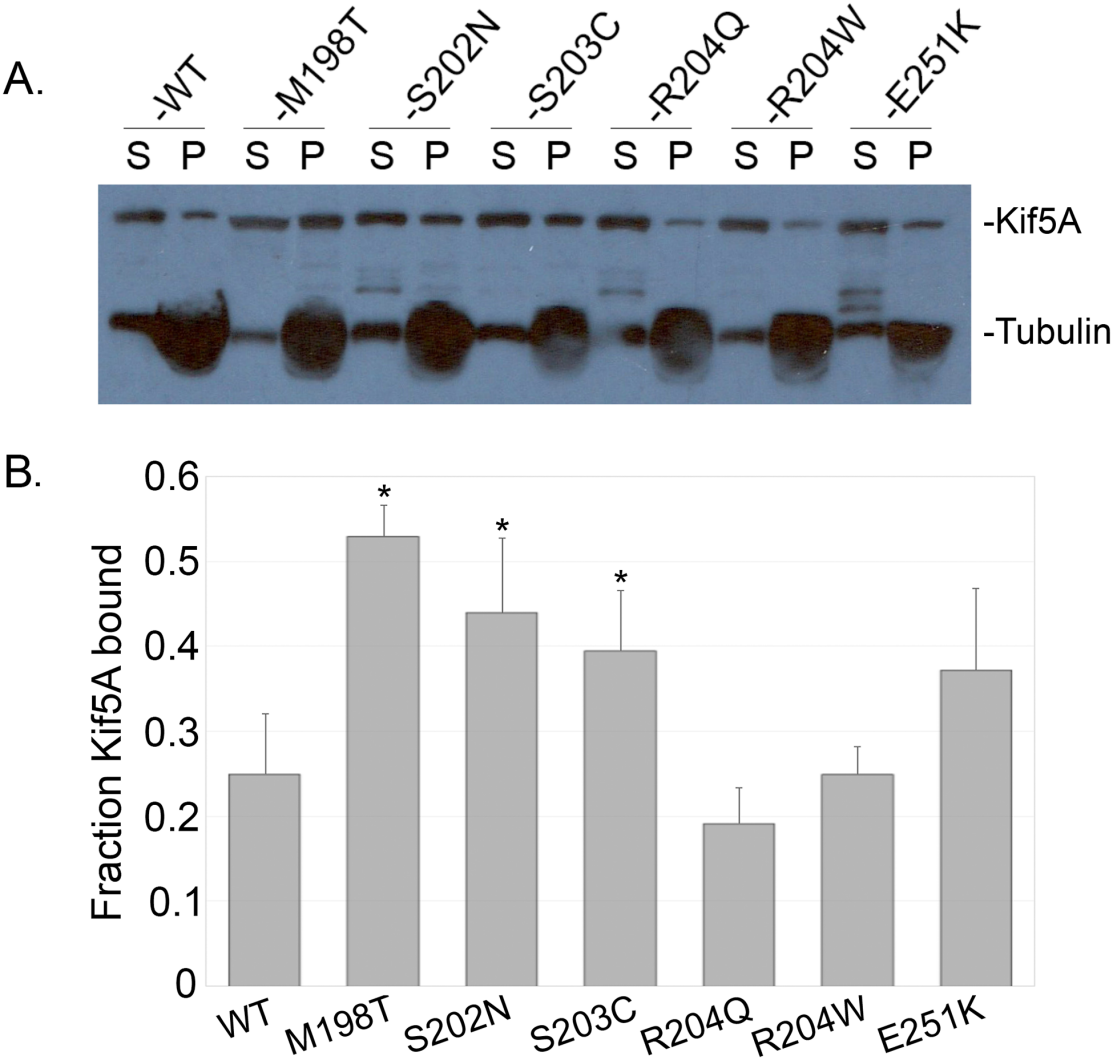
Microtubule affinity in the presence of ATP. A) Sample western blot of a single microtubule pelleting assay. S indicates the supernatant fraction and P indicates the pellet that had been resuspended in a volume of buffer equal to that of the supernatant. B) Quantitation of the average band densitometry of the pellet fraction from three separate microtubule pelleting assays for each Kif5A construct. Error bars show one standard deviation. Asterisks indicate a significant increase of kinesin in the microtubule-bound pellet fraction (p<0.05).

While we know that each of the mutants takes a significantly greater amount of time to progress all the way through the ATPase cycle (i.e. lower ATPase rates), we do not know which step in the cycle is slowing down the mutant’s catalytic activity. However, the differential affinity of kinesin for microtubules in the various substeps of the catalytic cycle may provide insight into this. In the absence of microtubules, the release of ADP from the nucleotide pocket is rate limiting for the catalytic cycle of conventional kinesin, while the interaction of kinesin with a microtubule significantly increases the rate of ADP release [33]. In the presence of microtubules and saturating amounts of ATP, kinesin releases from microtubules. Since this is a steady state assay, kinesin exists with highest probability in the species that precedes the rate-limiting step, which for kinesin must be either the release of phosphate or the release of ADP, as these are the two low affinity states (K-ADP-P_i_ and K-ADP). Thus, when wild type protein is incubated with microtubules and saturating ATP and the samples are spun at a rate in which microtubules, but not kinesin, will pellet we find kinesin largely in the supernatant (Fig 1A). If, however, there is a new rate-limiting step as a result of mutation, we may find a greater population of kinesin in one of the high affinity substeps (nucleotide-free or ATP bound). To test whether any of the mutants had a new rate-limiting step, we performed microtubule pelleting assays in which we incubated kinesin and microtubules in the presence of saturating ATP and spun them at 45,000 RPM. At this speed, microtubules will pellet and unbound kinesin will be found in the supernatant. However, if kinesin is bound to the microtubule (in a high affinity substep of the catalytic cycle) it will be dragged down into the pellet. We find that the M198T, S202N, and S203C mutants show a significantly greater percentage of the protein found in the microtubule-bound pellet fraction, suggesting that a greater percentage of the protein is in a high affinity substep of the catalytic cycle (representative western blot in Fig 1A and quantitation in Fig 1B).

### The R204Q/W and E251K mutants have a reduced microtubule affinity

**Fig 2:**
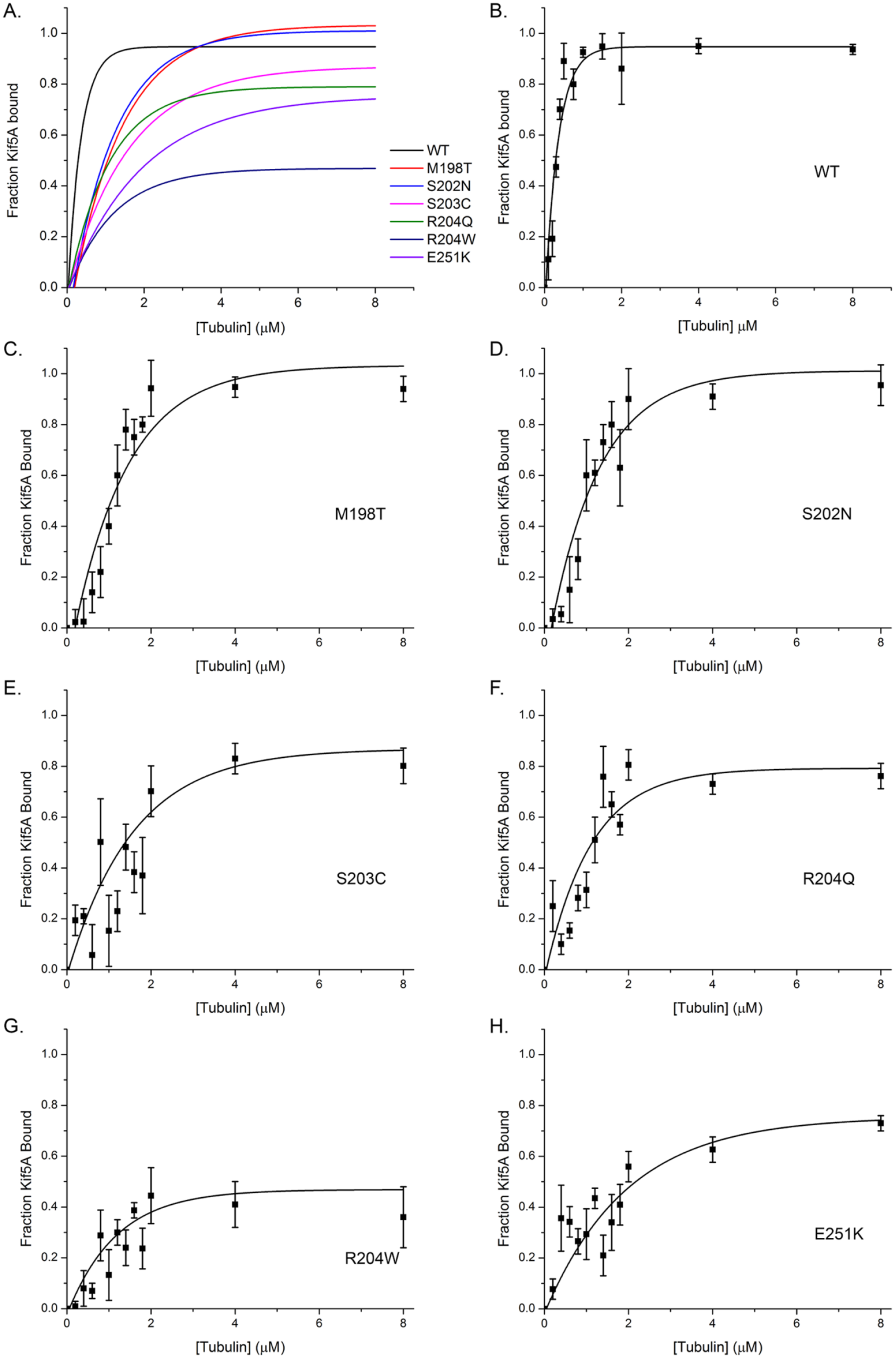
MT affinity in the presence of AMPPNP. MT pelleting assays were performed as described for Fig 1, except 5 mM AMPPNP was substituted in the reaction buffer. Experiments were repeated in a range of tubulin concentrations from 0 to 20 μM and the results of the densitometry analysis of the western blots produced are plotted for each Kif5A mutant. The curves shown are the best fit to the data using the exponential decay function in Origin 8 in a user free computational method. A) Curves of fraction Kif5A bound as a function of tubulin concentration for the wild type and mutant proteins. B-H) Individual Kif5A curves (WT or mutant as indicated) with mean +/- SD for each concentration of tubulin analyzed.

Conventional kinesin has a high affinity for microtubule in the presence of ATP. In the hand-over-hand model, the leading head is in the ATP bound state and must remain tightly bound to the microtubule to prevent detachment while the trailing head steps forward. However, because kinesin’s hydrolysis rate is so high, it is difficult to experimentally capture kinesin in this ATP-bound state. To test the affinity of the HSP mutant proteins in the ATP-bound state, we used the non-hydrolyzable ATP analog AMPPNP, which binds in the nucleotide pocket but can not be hydrolyzed by kinesin. We performed microtubule pelleting assays as described above, substituting 5 mM AMPPNP for 5 mM ATP. By performing these experiments at a variety of microtubule concentrations, we were able to generate the data shown in Fig 2. A 1 μM solution of wild type Kif5A protein saturates at nearly 100% protein bound at just under 2 μM tubulin. We note two interesting characteristics of the behavior of the HSP mutants. First, each of the mutants requires a greater tubulin concentration to reach saturation. Second, the R204Q, R204W, and E251K mutants plateau with a lower total fraction bound than wild type and the other mutants. This suggests that there is a fundamental change in microtubule affinity in the ATP-bound state that results from these HSP-causing mutations.

### Molecular dynamics simulations show that HSP mutations break an amino acid network

**Fig 3:**
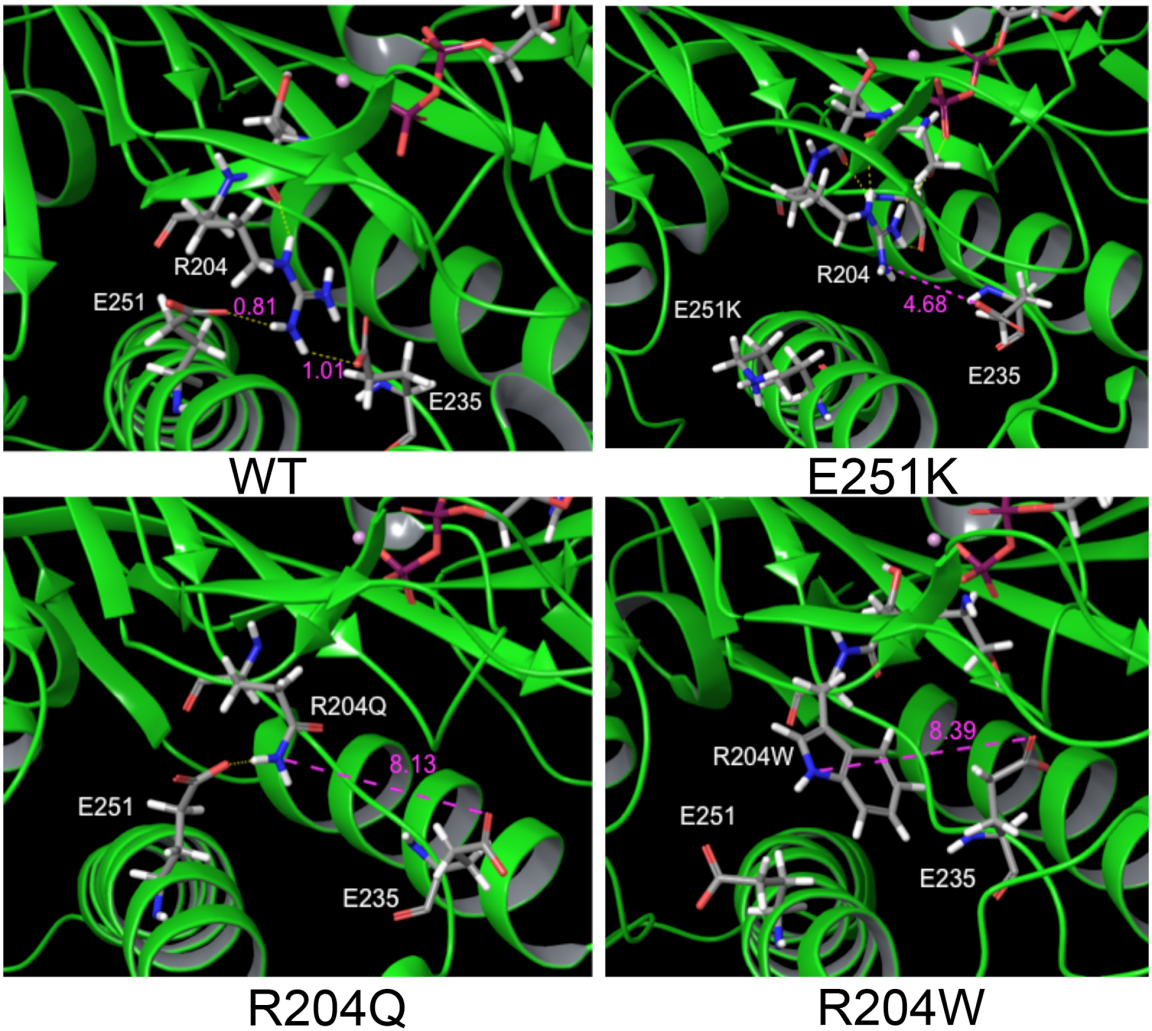
Molecular dynamics simulations of predicted altered kinesin structure. Images shown are the end point of a 10 ns simulation for wild type or mutant kinesin using the 4HNA structure as a starting point. Amino acids of interest are labeled in white. Intramolecular distances between potential bonding partners are shown in purple, with units of angstroms.

It has previously been shown that the absolutely conserved arginine (R204 in Kif5A) in Switch I forms a salt bridge with an absolutely conserved glutamate (E235 in Kif5A) that is necessary for the closure of the switches [36]. This interaction allows for the nucleophilic attack of the γ-phosphate bond by the catalytic water coordinated in the nucleotide pocket by residues in Switch I [23]. Mutating either of these residues in other kinesins leads to a significant reduction in the ATP hydrolysis rate [36]. To see what effect mutations in R204 might have on the structure of Kif5A in the ATP-bound state, we performed molecular dynamics simulations of the R204Q and R204W mutants by inserting the mutations into the published microtubule-bound crystal structure of conventional kinesin [27]. To test the validity of this process in observing the closed switch conformation, we first ran a simulation on the wild type protein (Fig 3, top left panel). We found that the amino group of R204 was in sufficiently close proximity to the carbonyl oxygen of E235 to interact (H-O distance of 1.01Å). When R204 is substituted with either glutamine or tryptophan, at the end of the simulation, we have lost this interaction with E235 (Fig 3, bottom panels). One other important aspect of the end of the wild type simulation was that in addition to its interaction with E235, the amino group of R204 is close enough to interact with the carbonyl of E251 (H-O distance of 0.81Å). Since the E251K mutant also showed reduced microtubule affinity in the microtubule pelleting assay, we ran a simulation with this mutant and found that the interaction with R204 was lost (Fig 3, top right panel). Perhaps more importantly, the loss of this E251-R204 interaction caused R204 to be significantly out of position, such that it could no longer interact with E235 (H-O distance of 4.68Å).

### HSP mutants have a reduced microtubule gliding velocity

While reduced enzymatic activity may be problematic for kinesin, the question remains whether or not this ultimately changes the motility characteristics of the kinesin motor. Our first hint that this may actually be the case was that only wild type motor showed single molecule motility in a TIRF-based assay (data not shown). Since microtubule gliding assays allow for the ensemble, collective behavior of multiple kinesins working together to generate motility, we performed microtubule gliding assays with our HSP mutant proteins. As shown in Table 3, two of the mutants showed significantly reduced microtubule gliding velocity, while the remainder showed no motility at all (although each retained the ability to recruit microtubules to the glass surface and remain stably bound). This shows that the enzymatic deficit associated with these HSP-causing mutations has a negative consequence on the kinesin’s motility.

**Table 3:** Microtubule gliding velocity.

Kif5A	Velocity (nm/sec)
Wild Type	486 +/- 35
M198T	54 +/- 6 *
S202N	non-motile
S203C	15.4 +/- 5.7 *
R204Q	non-motile
R204W	non-motile
E251K	non-motile

Data shown are the mean defined by the peak of a Gaussian fit to a histogram of the data. Data includes SD also generated from the Gaussian fit.

* indicates a significantly reduced velocity compared to wild type (p<0.01)

### Substitution of Mn^2+^ for Mg^2+^ gives a partial rescue of the S203C mutant

**Fig 4:**
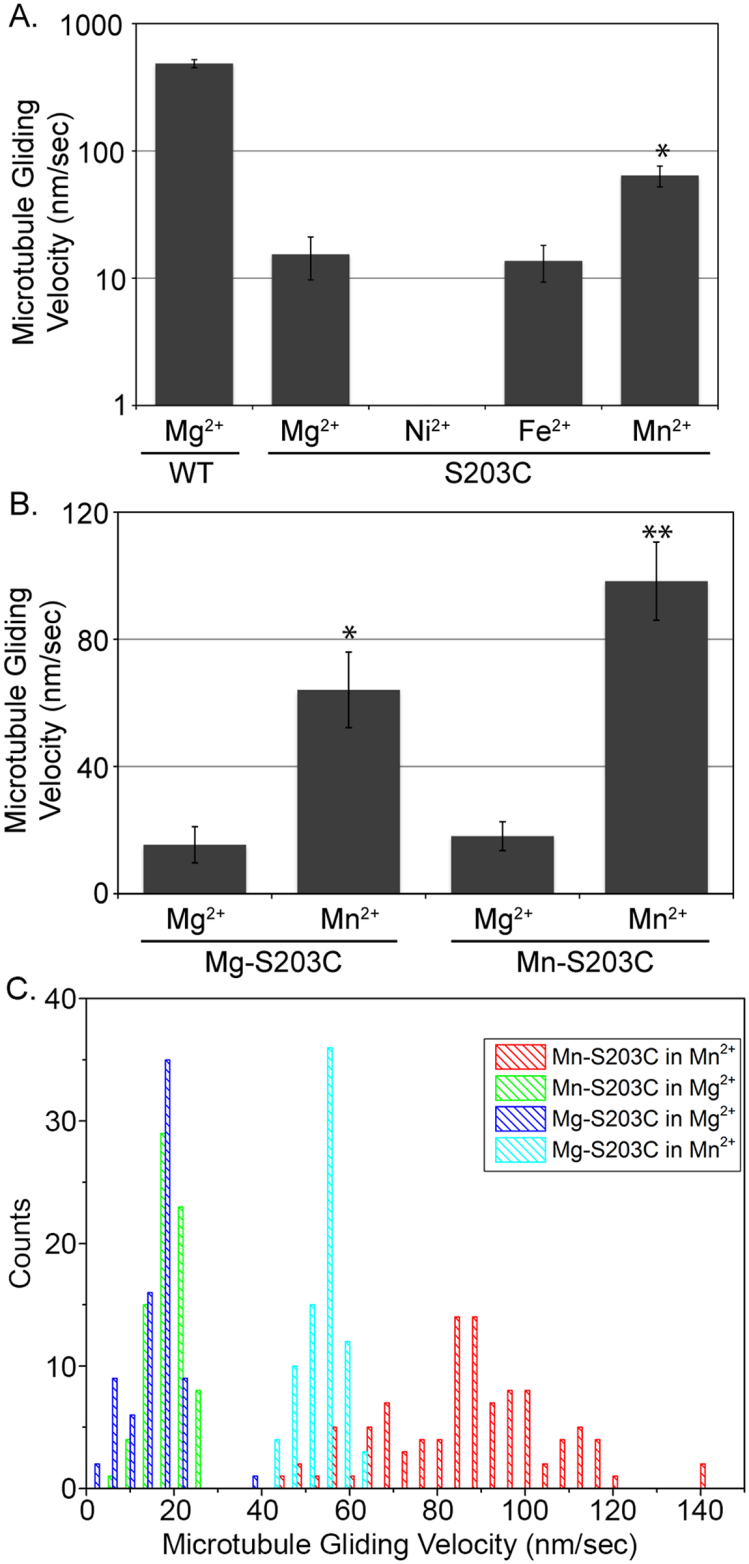
Effect of divalent cation on S203C microtubule gliding velocity. A) Microtubule gliding velocity of the S203C mutant in buffers with the divalent cations shown. The asterisk indicates that the velocity in Mn^2+^ is significantly greater than the velocity in Mg^2+^ (p<0.01), although still significantly lower than wild type in Mg^2+^ (p<0.01). Note the log scale of the Y-axis. B) Microtubule gliding velocity of the S203C mutant that was purified in the presence of Mg^2+^ (Mg-S203C) and Mn^2+^ (Mn-S203C). The single asterisk indicates that the velocity of Mg-S203C in Mn^2+^ is significantly greater than in Mg^2+^ (p<0.01), while the double asterisk indicates that the velocity of Mn-S203C in Mn^2+^ is significantly greater than in Mg^2+^ (p<0.01), and also significantly greater than the velocity of Mg-S203C in Mn^2+^ (p<0.05). C) Histograms of the data for S203C mutants in the conditions indicated. A similar number of microtubules (n = 90–110) were analyzed in each condition.

The second conserved serine in Switch I (NXXSSR) is necessary for the coordination of a catalytic Mg^2+^ by the side chain hydroxyl group that serves as an electron acceptor in ATP hydrolysis, and coordination via the backbone carbonyl of a catalytic water involved in nucleophilic attack of the γ-phosphate bond [23]. The HSP-causing S203C mutation swaps the Mg^2+^-coordinating hydroxyl group for a sulfhydryl group, which has been shown to be able to coordinate other divalent cations, such as Mn^2+^ and Zn^2+^ in similar enzymatic sites [37]. Indeed, Cochran and colleagues exploited this change in an effort to create a metal switch to control kinesin catalytic activity [38]. While this was a useful theoretical exercise, the finding that a similar mutation occurs in nature and causes a human heritable disease suggested that their findings could be translated to understanding and potentially treating patients with this mutation. We set out to test the hypothesis that the HSP-causing S203C mutation has a reduced activity in the presence of Mg^2+^, but that substitution of alternative divalent cations could modulate and potentially rescue its function. As shown previously in Table 3, the S203C mutant retains the ability to glide microtubules in an ensemble assay, although at a significantly reduced rate, ~30-fold slower than wild type. To test our hypothesis that alternative divalent cations could restore the activity of the S203C mutant, we repeated our microtubule gliding assays in identical buffers as our initial experiments, but substituted Ca^2+^, Cu^2+^, Fe^2+^, Mn^2+^, Ni^2+^, and Zn^2+^ for the Mg^2+^ in solution. Substitution of Ca^2+^ and Cu^2+^ caused microtubule disassembly and precipitation, respectively, so no data was obtained for these two cations. In the presence of Zn^2+^, the S203C mutant was unable to sufficiently interact with microtubules to recruit them from solution to the surface. When we substituted Ni^2+^ for Mg^2+^ in the buffer, microtubules were bound to the coverslip, but were completely immobile over the course of a 10-minute imaging series. However, we observed microtubule gliding by substitution of either Fe^2+^ or Mn^2+^ in the motility buffer. While microtubule motility by S203C was slower in the presence of Fe^2+^ than in the presence of Mg^2+^ (13.7 ± 4.4 nm/sec), there was a significant increase in the microtubule gliding rate by S203C upon substitution of Mn^2+^ (64.1 ± 11.9 nm/sec) (Fig 4A, S1A Fig).

While substitution of Mn^2+^ in our microtubule gliding assays led to a significant increase in the rate of microtubule gliding by the S203C mutant, the kinesin proteins had been purified in buffers that contained Mg^2+^, and not Mn^2+^. Early biochemical data from Taylor and colleagues suggests that chelation of divalent cation from the kinesin active site impairs the nucleotide binding ability of a large percentage of the purified protein [35, 39]. If mutation of the serine in the active site that normally coordinates Mg^2+^ to a cysteine reduces its binding capacity for the only divalent cation in solution, it could have a similar effect. To test this, we performed side-by-side purification of the S203C construct exclusively in the presence of Mg^2+^ (Mg-S203C) or Mn^2+^ (Mn-S203C) and used these proteins in microtubule gliding assays. As observed previously for Mg-S203C, there was a significant increase in the rate of microtubule gliding by Mn-S203C when Mn^2+^ was substituted into the motility buffer compared to motility in the presence of Mg^2+^ (Fig 4B, S1B Fig). However, there was also a significant increase in the rate of microtubule gliding by the Mn-S203C construct compared to the Mg-S203C construct in the presence of Mn^2+^ (Fig 4B).

**Fig 5:**
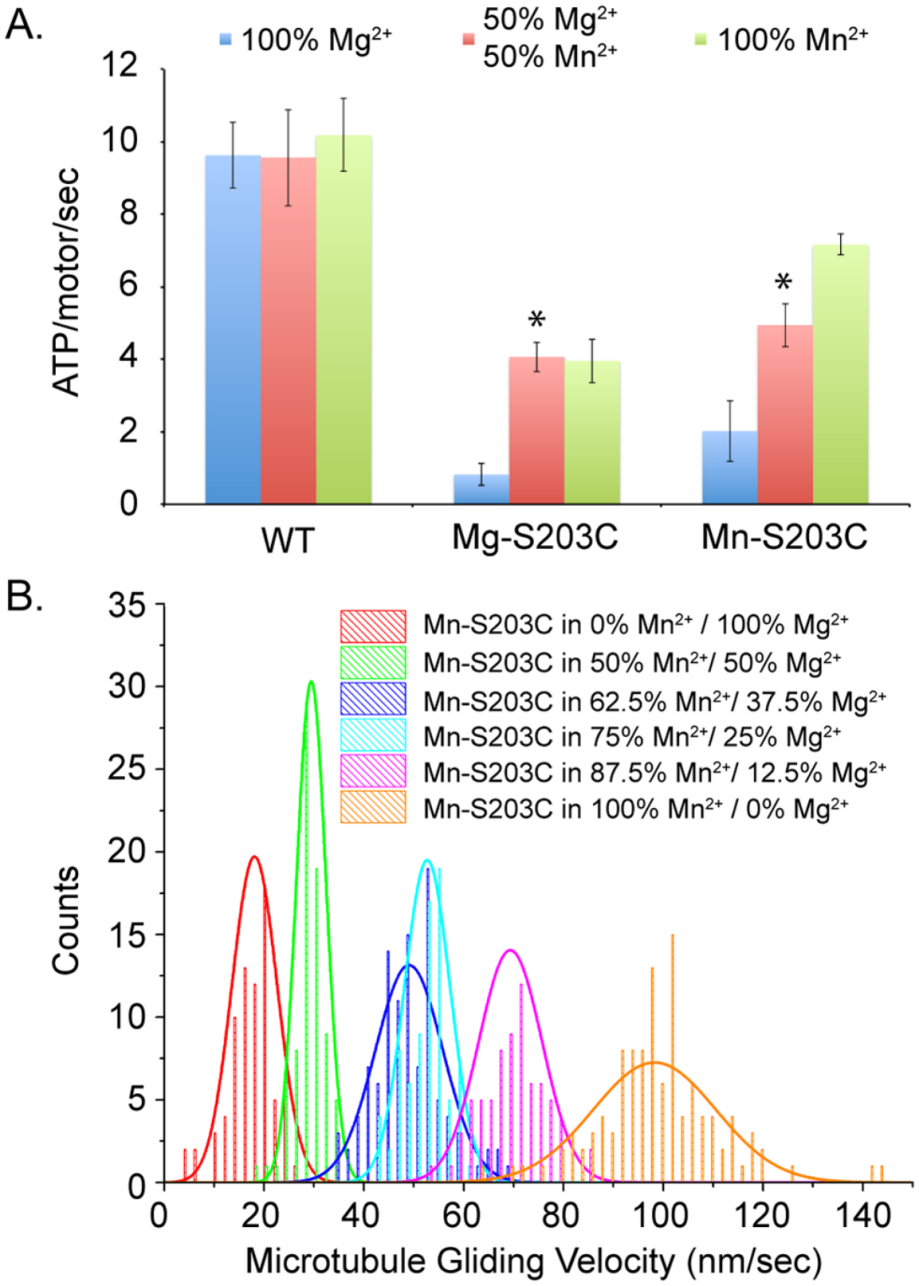
Mn^2+^-induced rescue of S203C activity. A) ATPase rate of WT, Mg-S203C, and Mn-S203C in buffers containing Mg^2+^ (100% Mg^2+^), Mn^2+^ (100% Mn^2+^), or an equal ratio of Mg^2+^ to Mn^2+^ (50% Mg^2+^, 50% Mn^2+^). The asterisk indicates that the ATPase rate in the 1:1 ratio of divalent cation is significantly greater than the rate in Mg^2+^ alone (p<0.01). B) Histograms with Gaussian fits to the data of Mn-S203C in buffer conditions with the relative Mn^2+^:Mg^2+^ ratios as shown.

As kinesin motility is inherently linked to its ATPase activity, we hypothesize that the reduced microtubule gliding rate is due to a reduction in the catalytic ATPase activity. To test this hypothesis, we performed microtubule-stimulated ATPase assays. In the presence of Mg^2+^, the activity of both Mg-S203C and Mn-S203C was significantly lower than wild type (Fig 5A), although Mn-S203C had a significantly greater rate than Mg-S203C in the presence of Mg^2+^ (p<0.01). To control for reduced ATPase activity due to working at sub-saturating tubulin concentration, we repeated the experiment at lower and higher tubulin concentrations and saw no significant change in rates (S2 Fig). We repeated the experiments in the presence of Mn^2+^, as well as in a buffer containing a 1:1 mixture of Mg^2+^ and Mn^2+^, keeping the overall ionic strength consistent (see experimental procedures for details). We found that a 1:1 mixture of Mg^2+^ and Mn^2+^ was sufficient to cause a significant increase in MT-stimulated ATPase activity (p<0.01), and that there was no additional significant benefit to completely substituting Mn^2+^ for Mg^2+^ in these assays (Fig 5A). Again, as we saw in the presence of Mg^2+^, the Mn-S203C construct had a significantly greater MT-stimulated ATPase activity (i.e. rescue) in both the 1:1 mixture and in Mn^2+^ alone. This suggests that the catalytic ATPase activity of the Mn-S203C protein is at least partially responsible for the increased microtubule gliding activity of Mn-S203C relative to Mg-S203C.

Our previous microtubule gliding assays were performed by complete substitution of one divalent cation for another. However, in ATPase assays we found that a 1:1 ratio of Mg^2+^ to Mn^2+^ in solution gave maximal MT-stimulated ATPase rescue. To determine whether this catalytic output (ATPase activity) translated into the ensemble mechanical output of the motor (microtubule gliding), we performed microtubule gliding assays using the Mn-S203C motor and motility buffer that contained a range of relative ratios of Mg^2+^:Mn^2+^ in solution, again keeping the overall ionic strength of the solution constant. As we increased the total percentage of Mn^2+^ in solution, we saw a corresponding dose-dependent increase in microtubule gliding velocity that peaked at 100% Mn^2+^ (Fig 5B). While there appears to be a disconnect between the results of the two assays (maximal rescue at 50%:50% in ATPase, dose-dependent to 100% Mn^2+^ in microtubule gliding), it is important to note that in addition to ATP binding and hydrolysis, microtubule gliding requires the requisite conformation changes in the motor domain and neck linker that enable directional motility and force generation. This could make the requirements for full rescue of the mechanical activity of the motor more stringent.

## Discussion

The current therapy to treat patients with HSP is a regimen of muscle relaxants and physical therapy. While this can be effective in helping to alleviate the manifestations of the disease, it addresses neither the mechanistic cause of the disease nor its progressive nature. Targeted therapeutics will require a fundamental understanding of the pathology behind the limb spasticity that all HSP patients share. This project is part of a larger effort to give mechanistic understanding to the disease such that the functional deficit associated with Kif5A mutations can be better understood [40], as well as the cellular loss of function that results [41, 42]. In particular, we have focused on the cluster of mutations in the Switch I motif to generate a mechanistic understanding of the role of this region as it relates to the functional deficit of Kif5A in the presence of HSP-causing mutations. We have shown that each mutation has a significant impact on at least one of the critical aspects of kinesin function, although there is variability in the severity of the decreased activity depending on the individual mutation.

### Catalytic deficits of Kif5A with HSP-causing mutations

We found that mutating residues in the conserved Switch I motif, known to play key roles in nucleotide coordination and hydrolysis, caused significant reductions in the basal and MT-stimulated ATPase rates of Kif5A. Yet while the binding of ATP, the hydrolysis of ATP, and the release of the hydrolyzed products is central to the function of kinesin, the nucleotide cycle is linked to other key properties of kinesin (altered microtubule affinity, force generation) that occur at sites in the protein distal to the nucleotide pocket. Likewise distal sites must relay information about the status of the protein (e.g. interacting with a microtubule) to change the catalytic activity of the motor. The networks of amino acid interactions that participate in these mechanotransduction events are poorly defined. It is interesting to note that while all Switch I mutants had a reduced basal rate of ATPase activity, the relative induction of increased ATPase activity of the M198T and S203C mutants was similar to that of wild type (~30-fold), albeit at lower rates. This suggests that although catalytic activity is reduced in these mutants, the mechanotransduction network that communicates microtubule binding to the nucleotide pocket is still intact. In the other mutants there was either no increase in ATPase activity (R204W) or a significantly reduced relative induction of ATPase activity compared to wild type (S202N at 5-fold, R204Q at 10-fold, and E251K at 6-fold). While it is tempting to attribute these amino acids as being necessary for this mechanotransduction network, we can not differentiate between this model and the possibility that the severity of the catalytic effect is such that the MT induction is limited. However, the molecular dynamics simulations suggest that two of these amino acids (R204 and E251) may be involved in this network.

### Mechanotransduction between the microtubule binding and nucleotide binding sites

Our molecular dynamics simulations of the R204 mutants and the E251K mutant were driven by the finding that all three mutants had a decreased affinity for microtubules. However, the simulations showed that all three were unable to form or stabilize a critical interaction between R204 and E235, necessary for nucleotide hydrolysis, not microtubule affinity. The finding that all three mutations also lost the connection between R204 and E235 may be just as important in the context of HSP. In conventional kinesin solutions structures, the alpha helix (α4) that runs between the alpha- and beta-tubulin subunits is significantly shorter, preceded by an extended Loop 11 [22]. However, both crystal structures of conventional kinesin bound to the alpha- and beta-tubulin heterodimer in the apo and ATP-bound form have an extended α4 helix that incorporates residues found in Loop 11 in the solution structures [26, 27]. While the functional significance of this helix extension is unknown, it is important to note that E251 is one of the Loop 11 residues in the solution structure that becomes ordered into the α4 helix in the tubulin-bound structures. While it is clear from the simulation involving the E251K mutant that the critical R204-E235 interaction is lost, there is also a significant reduction in microtubule affinity. This reduction in microtubule affinity is also seen in the R204Q and R204W mutants that lose the interaction with E251. These data suggest that the interaction between E251 and R204 that occur in the microtubule-bound state has dual function: stabilization of R204 in an orientation that is optimal for interaction with E235, and stabilization of the alpha helix and the coordination of microtubule binding. In this manner, the interaction with the microtubule is communicated to the nucleotide pocket and the status of the catalytic cycle is likewise communicated to the microtubule interface. Since the apo state in which kinesin has already formed the extended α4 helix occurs before the closure of the switches, it is likely that the primary communication is happening from the microtubule to the nucleotide pocket, but the details of this will ultimately have to be worked out. The fact that each of the mutants shows some amount of reduced binding to microtubules in the high affinity ATP-bound state (saturation at a higher concentration of tubulin, as well as a reduction in the total saturation stoichiometry in some mutants) suggests that this could be more difficult to parse with mutant analysis, although a recent paper by Cao and colleagues has used mutations that facilitate nucleotide release to solve solution structures of apo kinesin, showing two intermediate steps of microtubule association [43].

### Partial rescue of the S203C mutant with Mn^2+^

The Switch I motif has the dual function of coordinating the two nucleophilic waters in the nucleotide active site, as well as the divalent cation that acts as an electron acceptor in an intermediate step of γ-phosphate hydrolysis. Mutation of the conserved Mg^2+^-coordinating serine in Switch I to facilitate alternative metals was proposed as a potential “metal switch” to study molecular motor proteins [38]. Our work shows that in the case of one HSP-causing mutation, alteration of this conserved serine to a cysteine can be rescued with the alternative divalent cation Mn^2+^. We additionally found that the S203C mutant protein purified in the presence of Mn^2+^ had a significantly greater increase in the Mn^2+^-induced rescue effect than the corresponding protein purified in the presence of Mg^2+^. While we cannot rule out an allosteric effect of Mn^2+^ retained from purification, we have two potential interpretations for this result. First, the presence of Mn^2+^ during purification may help to stabilize the protein such that it retains its normal folded state. Since there is divalent cation and ATP in the purification buffers, basal ATPase activity (i.e. ATPase activity in the absence of microtubules) may be sufficient to alter the integrity of the protein when a sub-optimal cation is present. The other possibility is that the divalent cation may not be as labile as previously thought. If a subset of the S203C protein retains the original divalent cation from the purification, it would explain why the Mg-S203C does not see as significant a rescue in the presence of Mn^2+^. Since microtubule gliding assays and solution ATPase assays are readouts of ensemble behavior, we would expect a decrease in aggregate activity due to any protein that still has its original Mg^2+^ ion bound to the active site. While we find this idea intriguing, it is beyond the scope of this present work.

This work represents the first characterization of a mutation in the conserved Switch I motif that causes hereditary spastic paraplegia. Although the serine in this motif that coordinates the active site Mg^2+^ is absolutely conserved in kinesin transport motors in humans, we find that the S203C mutant still retains catalytic activity, albeit at significantly reduced levels. We have also shown a dose-dependent rescue in the catalytic function of the S203C mutant protein by substituting Mn^2+^ for Mg^2+^. While it is tempting to postulate a potential therapeutic for patients based on these results, our understanding of the cellular uptake mechanism of manganese is incomplete at best, and there is significant debate about the cellular concentration of free manganese. Minimally, we have provided mechanistic insight into the basis of the disease patients with this mutation.

### A transport deficit at the heart of HSP caused by Kif5A mutation?

All of the mutations in Kif5A tested in this work cause the same disease in humans—HSP. This happens even though the deficit in catalytic activity from one mutant to the next shows a continuum of changes. However, what they all share in common is either a loss of, or a significant deficit in, motility. Thus, it is logical to think that the neurodegenerative nature of this axonopathy at the heart of HSP is due to a deficit of transport. If a critical factor that is normally transported by Kif5A is no longer reaching the axon terminus (at all or in high enough concentration), the synaptic connection to its target may no longer be retained. Future studies in this laboratory are aimed at identifying the subset of axonal cargoes that are transported by Kif5A, and determining which of them show altered axonal transport kinetics in the presence of HSP-causing mutations in Kif5A.

## Supporting information

S1 Fig Kymographs of S203C mutant motility. Kymographs were generated using the ImageJ reslice feature. A maximum Z-projection of an image stack from an acquired time-lapse series was made and a line was drawn along the length of a microtubule in the maximum projection. The resulting kymograph is shown in this figure, where the slope of the line is the velocity of the microtubule.
(TIF)

S2 Fig ATPase rates with increasing microtubule concentration. To test whether we had added a sufficient number of microtubules to maximize the ATPase rate of each construct, we performed the assays in a concentration series of tubulin as shown. We see no significant difference in the ATPase rate of any of the three conditions when increasing the tubulin concentration from 1 μM tubulin to 3 μM tubulin.
(TIF)
