# Supplementary figures and images for: Characterization of kinesin switch I mutations that cause hereditary spastic paraplegia
Source: PLoS One. 2017 Jul 5;12(7):e0180353. doi: 10.1371/journal.pone.0180353 (PMC5498027; doi:10.1371/journal.pone.0180353)

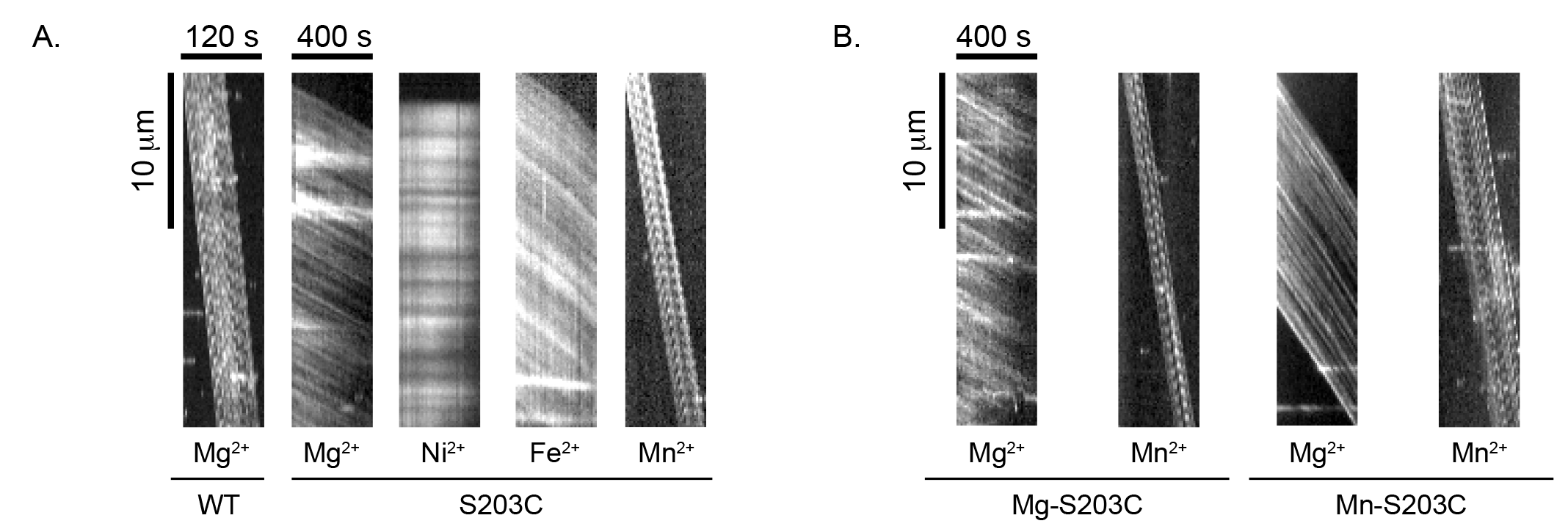

Supplement: S1 Fig — Kymographs were generated using the ImageJ reslice feature. A maximum Z-projection of an image stack from an acquired time-lapse series was made and a line was drawn along the length of a microtubule in the maximum projection. The resulting kymograph is shown in this figure, where the slope of the line is the velocity of the microtubule. (TIF) [file pone.0180353.s001.tif]

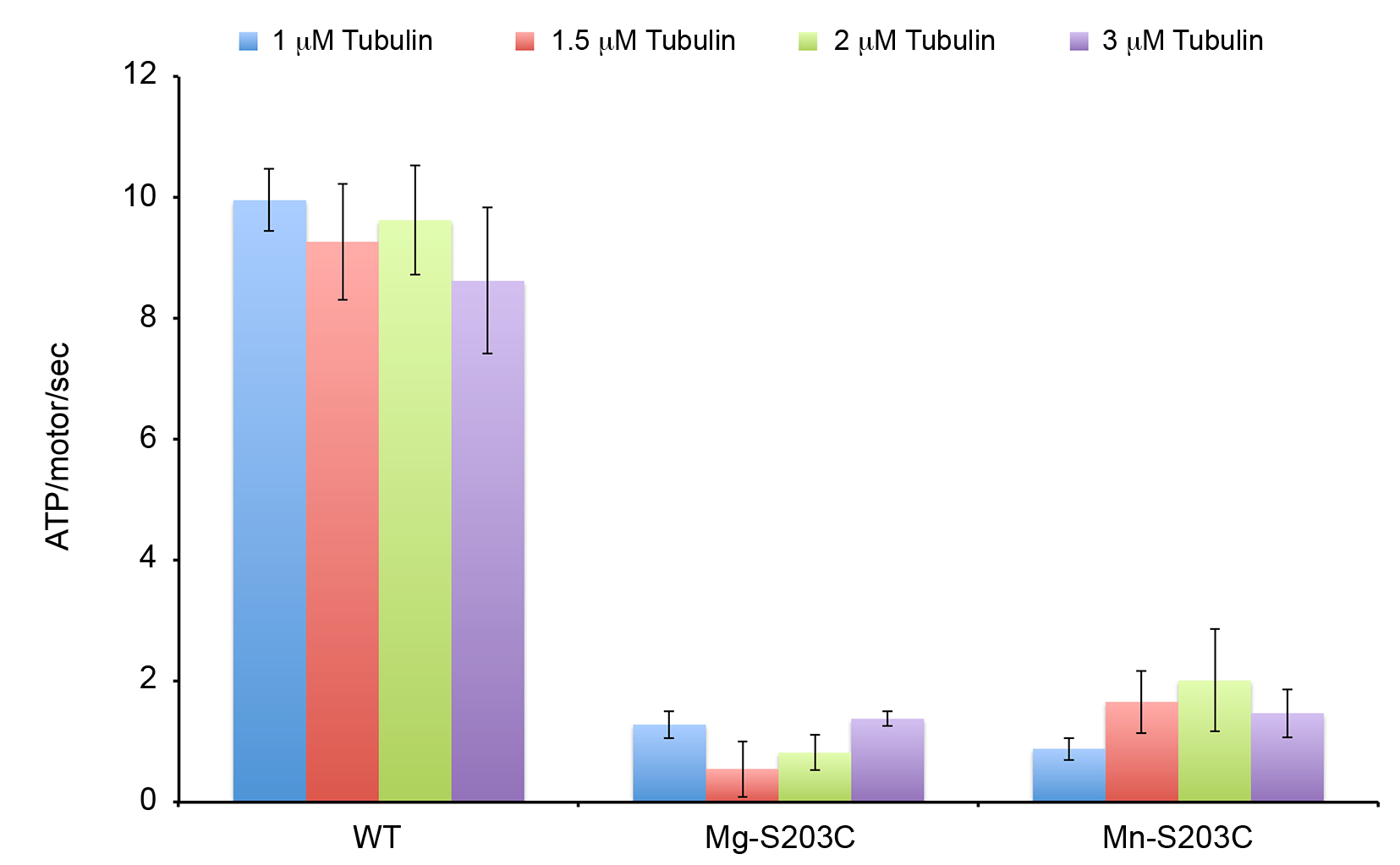

Supplement: S2 Fig — To test whether we had added a sufficient number of microtubules to maximize the ATPase rate of each construct, we performed the assays in a concentration series of tubulin as shown. We see no significant difference in the ATPase rate of any of the three conditions when increasing the tubulin concentration from 1 μM tubulin to 3 μM tubulin. (TIF) [file pone.0180353.s002.tif]
